# Occupation and skin cancer: the results of the HELIOS-I multicenter case-control study

**DOI:** 10.1186/1471-2458-7-180

**Published:** 2007-07-26

**Authors:** Berta Suárez, Gonzalo López-Abente, Carmen Martínez, Carmen Navarro, Maria José Tormo, Stefano Rosso, Simon Schraub, Lorenzo Gafà, Hélène Sancho-Garnier, Janine Wechsler, Roberto Zanetti

**Affiliations:** 1 Environmental and Cancer Epidemiology Unit, National Center for Epidemiology, Carlos III Institute of Health, Sinesio Delgado 6, 28029 Madrid, Spain; 2 Andalusian School of Public Health, Granada, Spain; 3 Directorate-General for Public Health, Murcia, Spain; 4 Registro Tumori Piemonte, Turin, Italy; 5 Centre de lutte contre le cancer Paul Strauss, Université Luis Pasteur, 67000 Strasbourg, France; 6 Lega Italiana per la lotta contro I tumori Sezione provinciale Ragusa. Ragusa, Italy; 7 Epidaure, Montpellier, France; 8 Service d'Anatomie et de Cytologie Pathologiques, Hospital Henry Mondor, 94010 Créteil, France

## Abstract

**Background:**

Non-melanoma skin cancer (NMSC) is the most frequent tumour among Caucasian populations worldwide. Among the risk factors associated with this tumour, there are host-related factors and several environmental agents. A greater likelihood of high exposure to physical agents (with the exception of solar radiation) and chemical agents depends on the work setting. Our objective is to evaluate the role of occupational exposures in NMSC, with special emphasis on risk factors other than solar radiation and skin type.

**Methods:**

We analysed 1585 cases (1333 basal cell carcinoma (BCC) and 183 squamous cell carcinoma (SCC)) and 1507 controls drawn from the Helios-I multicenter study. Odds ratios (OR) and 95% confidence intervals (CI) were estimated using logistic regression mixed models.

**Results:**

For NMSC as a whole (both *histological types*), miners and quarrymen, secondary education teachers, and masons registered excess risk, regardless of exposure to solar radiation and skin type (OR 7.04, 95% CI 2.44–20.31; OR 1.75, 95% CI 1.05–2.89 and OR 1.54, 95% CI 1.04–2.27, respectively). Frequency of BCC proved higher among railway engine drivers and firemen (OR 4.55; 95% CI 0.96–21.57), specialised farmers (OR 1.65; 95% CI 1.05–2.59) and salesmen (OR 3.02; 95% CI 1.05–2.86), in addition to miners and quarrymen and secondary education teachers (OR 7.96; 95% CI 2.72–23.23 and OR 1.76; 95% CI 1.05–2.94 respectively). The occupations that registered a higher risk of *SCC (though not of BCC*) were those involving direct contact with livestock, construction workers not elsewhere classified (OR 2.95, 95% CI 1.12–7.74), stationary engine and related equipment operators not elsewhere classified (OR 5.31, 95% CI 1.13–21.04) and masons (OR 2.55, 95% CI 1.36–4.78).

**Conclusion:**

Exposure to hazardous air pollutants, arsenic, ionizing radiations and burns may explain a good part of the associations observed in this study. The Helios study affords an excellent opportunity for further in-depth study of physical and chemical agents and NMSC based on matrices of occupational exposure.

## Background

Non-melanoma skin cancer is the most frequent tumour among Caucasian populations worldwide. Nevertheless, the study of its frequency poses difficulties. As one of the basic data sources for identification of cases in population cancer registries are hospital and pathology records, the fact that non-melanoma skin cancer is often not grounds for hospital admission may well lead to under-registration of cases. Furthermore, being a disease that can progress with few symptoms and is basically manifested in persons of advanced age, it may never be diagnosed. Cure rates stand at around 99% [1], with the result that it is a tumour to which relatively little attention is paid and is often not included among cancers targeted by population cancer registries. Variability in incidence rates is very marked, something that might in part be due to greater or lesser comprehensiveness of the case registry, as well as differences in risk among populations. The highest European incidence rates correspond to cancer registries in Ireland and Geneva (Switzerland), with rates close on 100 per 100,000 population [2]. In surveys conducted in Australia, annual incidence rates were estimated to exceed 1,000 per 100,000 population [3,4].

In all, 80–85% of non-melanoma skin cancers are basal cell carcinomas (BCC) and the remaining percentage are squamous cell carcinomas (SCC), with the latter being the more invasive of the two and underlying most of the deaths attributable to these tumours [5]. Both histologic types consistently register a positive relationship with exposure to solar ultraviolet (UV) radiation and an inverse relationship with the degree of skin pigmentation in the population [6], though differences nevertheless do exist between the two histologic types in terms of risk of presenting with cancer by type of exposure (brief-intense in basal cell, and prolonged-accumulated in squamous cell carcinomas) [7]. Among the risk factors associated with this tumor, there are host-related factors, such as skin pigmentation, precursor lesions (actinic keratosis or Bowen's disease), genetic predisposition, and immunologic factors. **Physical agents **(ultraviolet and ionizing radiations) [8-11] and **chemical agents **(polycyclic aromatic hydrocarbons, arsenic, and nitrosamines) [12-15], as well as diet-related factors, viruses and the predisposition generated by certain traumas, burns and scars have been identified as environmental etiologic agents [16-18].

A greater likelihood of high exposure to physical agents (with the exception of solar radiation) and chemical agents depends on the work setting. Hence, this study sought to evaluate the role of occupational exposures in non-melanoma skin cancer, with special emphasis on risk factors other than solar radiation, using data drawn from the Helios-I multicenter study for the purpose [7,8]. Such additional factors are connected with exposures to environmental chemical substances (e.g., chimney soot, arsenic compounds, polycyclic hydrocarbons), chronic skin irritation, viral infections, and immune factors that can predispose to this type of cancer [19-23].

## Methods

The Helios I study was a European multicenter case-control study. Its design is briefly outlined below, with a more detailed description to be found in the reference section [7,8]

### Selection of cases

We included all cases of non-melanoma skin cancer registered from November 1989 through June 1993 in the following 6 European regions: Turin (north-west Italy); Ragusa (Sicily); Trento (North-East Italy); Villejuif and Créteil (Paris); Besançon (Franche-Comté, France); Murcia (south-east Spain); and Granada (Andalusia, Spain). In Turin, Ragusa, Besançon, Murcia and Granada, population cancer registers that covered a total population of 3.5 million were used as the case source. In these areas, all incident cases aged 20 to 70 years with diagnosis of BCC, SCC or skin carcinoma identified by the reporting systems, were deemed eligible. In Paris, case data were collected at two specialist centers, the Gustave-Roussy Institute in Villejuif and the Henri Mondor Hospital in Créteil. Dermatologists as well as general practitioners asked cases for their consent to being interviewed on lifestyle and health. In population-based centers, cases were interviewed at the dermatology clinic itself or at home, whereas at hospital-based centers, they were interviewed during their stay in hospital. Morphologic diagnoses were validated by a panel of pathologists who carried out a blind review of the biopsies [24].

### Selection of controls

The group of controls was obtained by random sampling, duly stratified by age and sex, and conducted in the same regions in which the cases were recruited. The strata were proportional to the distribution of cases by age and sex. The sample was recruited on the basis of electoral censuses in Ragusa and Besançon and municipal rolls in Turin, Murcia and Granada. In the case of Paris, the controls were obtained by means of random sampling, based on hospital registers and excluding all patients with cancer or skin diseases. Controls were contacted by letter, and interviewed at home, in the workplace, or at the cancer registry. In the case of hospital controls, such interviews took place during their stay in hospital.

### Assessment of exposure

Questionnaires were completed during an interview conducted by purpose-trained staff. A section of the questionnaire recorded information on participants' work history, i.e., any job held during their lifetime with a minimum duration of 6 months. They were asked about the type of work, the firm's activity, and the starting and finishing dates. In addition, this section included questions on outdoor work performed, hours per day and months worked in the periods May-September or October-April, whether subjects worked partly unclothed, whether they wore a head covering and, lastly, whether they wore socks or stockings during work done in summer. Another sections reported on participants' use of leisure time, and on their phenotype characteristics.

Occupations were classified and coded according to the International Standard Classification of Occupations (ISCO) [25]. Analyses were performed for 10 major occupational groups, defined by the first of the four digits code, and for occupations defined by the first three digits of the code. We selected a total of 157 three-digit occupations having a minimum of five exposed individuals with at least one case and one control.

Insofar as the "exposure to sun" variable was concerned, this was measured on a continuous scale, as total hours, in terms of solar exposure during vacations and during outdoor work, weighted and not weighted by season of exposure (on average, solar irradiation in summer is double that in winter). Quartiles were calculated and the "exposure to sun" variable categorized on this basis. A complete explanation of sun exposure recording can be consulted on Rosso et al. [7].

In addition, the models also included variables that had proven to be independent risk indicators in previous analyses [7,8,26], namely: color of eyes; natural hair color at age 20 years; and reaction of skin to solar exposure (history of sunburn). References to "phenotype" in the text are to these three components.

In the analysis, we assessed the effect of occupation on appearance of basal cell and squamous cell carcinomas, considered jointly and singly. ORs and their 95% confidence intervals (CI) were calculated using unconditional logistic regression mixed models. In a first analysis, estimates were adjusted for age and sex (variables matched by frequency), with exposure to solar radiation and phenotype being added subsequently. Center/town was included as a random effects term in all analyses [27].

At the time the study has been conducted ethical approval was not required for epidemiological studies in none of the involved countries. Written consent was obtained from every recruited subject, in order to both analyzing the data acquired and accessing the relevant diagnostic documents (e.g. pathology reports).

## Results

The participation rate, as described elsewhere [8] was 85.8% among cases and 69.3% among controls. For this analysis, we included 3092 participants, with 1585 cases and 1507 controls. We excluded 297 cases and 288 controls since information on occupation was not available. Among the cases, 1333 presented with basal cell, and 183 with squamous cell carcinoma. Mean age was 60.5 years for cases and 58.2 years for controls. A total of 63% of cases and 62% of controls were men. Shown in Table 1 is the breakdown of the study by participant region, from which it will be seen that Turin was the region with the greatest contribution to the study, followed by Granada, Murcia and Besançon.

**Table 1:** Number of persons included in this analysis, by center and histologic type

**Center**	**Total number**	**Cases**	**BCC**	**SCC**	**Controls**
Granada	626	310	263	33	316
Murcia	548	295	228	57	253
Besançon	495	247	203	28	248
Villejuif	196	98	82	11	98
Créteil	190	95	80	12	95
Turin	829	432	400	24	397
Ragusa	208	108	77	18	100

Total	3092	1585	1333	183	1507

BCC: basal cell carcinomas

SCC: squamous cell carcinomas

In 69 cases there was no record of histologic type

Table 2 shows the results of the analysis by major occupational group, defined by the first digit of the ISCO code, for: a) all cases; b) basal cell carcinomas; and, c) squamous cell carcinomas. Two effect estimates are shown, one adjusted for age and sex, and the other additionally adjusted for solar exposure and phenotype. Both estimates are adjusted for center as a random effects term (amounting to a conservative constraint). The analysis by major group yielded no statistically significant association, save for group 0 (professional, technical and related workers) in the case of basal cell carcinomas. However, this association became attenuated after adjustment for solar exposure and phenotype, and failed to attain significance. In the case of squamous cell carcinomas, the groups classified as clerical and related workers and sales workers registered a significantly lower risk than did the remaining occupations.

**Table 2:** Effect of occupation (major groups) on non-melanoma skin cancers. OR and 95% confidence intervals. Estimates adjusted for a) age group, sex and center; b) age group, sex, exposure to sun, skin type and center. All cases, basal cell and squamous cell

		TOTAL CASES					
		
				Adjusted for age and sex		Adjusted for age, sex, exposure to sun, and skin type	
				
		Exp	No exp	OR	95% CI	OR	95% CI
Physical scientists, architects and engineers, biological and health scientists, mathematicians and economists	Cases	129	1455	1.28	0.98–1.69	1.21	0.91–1.62
	Controls	98	1407				
Accountants, jurists, teachers, writers and artists	Cases	185	1400	1.13	0.90–1.41	1.11	0.88–1.40
	Controls	158	1349				
Administrative and managerial workers	Cases	60	1524	1.32	0.88–1.97	1.30	0.86–1.96
	Controls	43	1464				
Clerical and related workers	Cases	287	1295	0.93	0.77–1.11	0.88	0.73–1.07
	Controls	292	1212				
Sales workers	Cases	176	1406	0.90	0.72–1.13	0.89	0.71–1.11
	Controls	185	1321				
Service workers	Cases	283	1302	0.85	0.71–1.02	0.85	0.71–1.02
	Controls	308	1196				
Agriculture, animal husbandry and fishermen	Cases	486	1097	1.06	0.91–1.25	1.18	0.96–1.45
	Controls	436	1069				
Miners, metalworker, woodworkers, chemical workers	Cases	288	1297	1.08	0.90–1.30	1.12	0.93–1.35
	Controls	258	1248				
Leather workers, welders, electricians and glass workers	Cases	289	1295	0.91	0.76–1.10	0.90	0.74–1.08
	Controls	295	1211				
Rubber workers, graphic artists, painters, builders, transport workers	Cases	431	1153	1.08	0.91–1.28	1.10	0.93–1.31
	Controls	387	1118				

		BASAL CELL					

Physical scientists, architects and engineers, biological and health scientists, mathematicians and economists	Cases	115	1217	1.38	1.04–1.83	1.26	0.94–1.69
	Controls	98	1407				
Accountants, jurists, teachers, writers and artists	Cases	152	1181	1.10	0.87–1.40	1.07	0.84–1.37
	Controls	158	1349				
Administrative and managerial workers	Cases	50	1282	1.35	0.89–2.05	1.31	0.85–2.01
	Controls	43	1464				
Clerical and related workers	Cases	253	1077	0.97	0.80–1.17	0.90	0.74–1.10
	Controls	292	1212				
Sales workers	Cases	159	1172	0.98	0.78–1.22	0.96	0.76–1.21
	Controls	185	1321				
Service workers	Cases	246	1087	0.88	0.73–1.06	0.88	0.73–1.07
	Controls	308	1196				
Agriculture, animal husbandry and fishermen	Cases	394	939	1.04	0.88–1.23	1.21	0.97–1.50
	Controls	436	1069				
Miners, metalworker, woodworkers, chemical workers	Cases	246	1087	1.10	0.90–1.33	1.14	0.93–1.39
	Controls	258	1248				
Leather workers, welders, electricians and glass workers	Cases	250	1083	0.96	0.79–1.16	0.93	0.76–1.13
	Controls	295	1211				
Rubber workers, graphic artists, painters, builders, transport workers	Cases	353	980	1.07	0.90–1.27	1.10	0.92–1.32
	Controls	387	1118				

		SQUAMOUS CELL					

Physical scientists, architects and engineers, biological and health scientists, mathematicians and economists	Cases	10	173	0.88	0.45–1.72	0.85	0.40–1.80
	Controls	98	1407				
Accountants, jurists, teachers, writers and artists	Cases	16	167	0.81	0.46–1.43	0.80	0.43–1.48
	Controls	158	1349				
Administrative and managerial workers	Cases	8	175	1.27	0.59–2.77	1.43	0.61–3.32
	Controls	43	1464				
Clerical and related workers	Cases	19	164	0.55	0.34–0.90	0.58	0.34–0.99
	Controls	292	1212				
Sales workers	Cases	12	170	0.48	0.26–0.88	0.48	0.25–0.92
	Controls	185	1321				
Service workers	Cases	24	159	0.66	0.42–1.02	0.65	0.40–1.03
	Controls	308	1196				
Agriculture, animal husbandry and fishermen	Cases	75	106	1.34	0.95–1.89	1.00	0.65–1.54
	Controls	436	1069				
Miners, metalworker, woodworkers, chemical workers	Cases	30	153	1.01	0.67–1.53	1.13	0.73–1.75
	Controls	258	1248				
Leather workers, welders, electricians and glass workers	Cases	30	152	0.72	0.47–1.10	0.79	0.51–1.23
	Controls	295	1211				
Rubber workers, graphic artists, painters, builders, transport workers	Cases	62	120	1.21	0.87–1.69	1.19	0.83–1.70
	Controls	387	1118				

Exp : Exposed

No Exp : No exposed

Tables 3 and 4 show the analysis for the three-digit occupations. We analyzed a total of 157 occupations that had a minimum number of exposed subjects, but Table 3 only lists the results of occupations that displayed statistical significance and/or an OR of 2 or higher.

**Table 3:** Non-melanoma skin cancer. OR and 95% confidence intervals associated with selected occupations*

		Cases		Controls		Adjusted for age and sex		Adjusted for age, sex, and exposure to sun		Adjusted for age, sex, exposure to sun, and skin type	
			
Code	ISCO	Exp	No Exp	Exp	No Exp	OR	95% CI	OR	95% CI	OR	95% CI
034	Engineering Technicians	6	1579	3	1504	1.90	0.47 – 7.60	1.86	0.46 – 7.49	2.07	0.51 – 8.44
067	Pharmacist	6	1579	2	1505	2.88	0.58 – 14.36	2.81	0.56 – 14.02	2.82	0.55 – 14.43
132	Secondary Education Teachers	46	1539	25	1482	**1.80**	**1.10 – 2.95**	**1.78**	**1.08 – 2.92**	**1.75**	**1.05 – 2.89**
193	Social Workers	5	1580	2	1505	2.47	0.48 – 12.79	2.41	0.46 – 12.47	2.25	0.42 – 12.16
212	Factory Managers	9	1576	2	1505	4.37	0.94 – 20.33	4.24	0.91 – 19.74	3.46	0.73 – 16.33
342	Computer Operators	7	1578	2	1505	3.52	0.73 – 17.01	3.46	0.71 – 16.77	3.07	0.62 – 15.22
399	Clerks n.e.c.	6	1579	3	1504	1.97	0.49 – 7.93	1.96	0.49 – 7.86	2.08	0.50 – 8.59
431	Sales Engineers	13	1572	5	1502	2.49	0.88 – 7.01	2.44	0.87 – 6.89	2.53	0.88 – 7.25
531	Cooks	8	1577	25	1481	0.31	0.14 – 0.69	0.31	0.14 – 0.69	0.34	0.15 – 0.76
551	Building Caretakers	12	1573	23	1484	0.50	0.25 – 1.00	0.50	0.25 – 1.00	0.46	0.23 – 0.95
612	Specialized Farmers	55	1530	36	1471	1.44	0.94 – 2.21	1.47	0.95 – 2.26	1.49	0.96 – 2.32
711	Miners and Quarrymen	29	1556	4	1503	**6.86**	**2.40 – 19.6**	**7.07**	**2.47 – 20.24**	**7.04**	**2.44 – 20.31**
728	Galvinizers	11	1574	4	1503	2.60	0.82 – 8.20	2.58	0.82 – 8.16	2.91	0.91 – 9.25
749	Chemical Worker s	5	1580	1	1506	4.58	0.53 – 39.39	4.59	0.53 – 39.46	4.51	0.51 – 39.87
773	Butchers and Meat Preparers	8	1577	17	1489	0.45	0.19 – 1.04	0.44	0.19 – 1.04	0.41	0.17 – 0.97
811	Cabinetmakers	3	1582	10	1497	0.28	0.08 – 1.02	0.28	0.08 – 1.02	0.27	0.07 – 0.99
855	Electricians	9	1576	20	1487	0.42	0.19 – 0.94	0.42	0.19 – 0.93	0.38	0.17 – 0.85
926	Bookbinders	7	1578	3	1504	2.26	0.58 – 8.77	2.26	0.58 – 8.77	2.12	0.53 – 8.46
951	Masons	69	1516	47	1460	1.42	0.97 – 2.08	1.44	0.98 – 2.13	**1.54**	**1.04 – 2.27**
983	Railway Engine Drivers and Firemen	10	1575	2	1505	4.55	0.99 – 20.89	4.49	0.98 – 20.62	4.14	0.89 – 19.29
999	Laborers n.e.c.	122	1463	90	1417	1.32	0.99 – 1.76	**1.34**	**1.00 – 1.80**	**1.37**	**1.02 – 1.85**

* Criterion: OR>2 or statistically significant OR (lower limit of OR adjusted for age, sex, exposure to sun and skin type > = .9 or upper limit <1).

**Table 4:** Basal cell (BCC) and squamous cell carcinoma (SCC). OR and 95% confidence intervals associated with each occupation

				BASAL CELL					SQUAMOUS CELL			
			
		Controls	BCC	Adjusted for age and sex		Adjusted for age, sex, exposure to sun, and phenotype		SCC	Adjusted for age and sex		Adjusted for age, sex, exposure to sun, and phenotype	
			
		1507	1333					183				
		
CODE	ISCO	Exp	Exp	OR	95% CI	OR	95% CI	Exp	OR	95% CI	OR	95% CI
023	Electrical Engineers	3	4	1.50	0.34 – 6.74	1.10	0.24 – 5.05	1	2.93	0.30 – 29.16	3.24	0.29 – 35.73
033	Surveyor's Assistants	4	2	0.58	0.11 – 3.18	0.46	0.08 – 2.64	1	2.13	0.24 – 19.10	2.11	0.21 – 21.23
039	Engineer's Aides	5	2	0.46	0.09 – 2.38	0.45	0.08 – 2.36	1	1.81	0.22 – 15.06	2.47	0.23 – 26.27
067	Pharmacists	2	4	2.17	0.39 – 11.89	1.98	0.34 – 11.44	1	6.29	0.46 – 86.66	7.26	0.49 – 108.42
111	Professional Accountants	42	34	0.92	0.58 – 1.48	0.99	0.61 – 1.60	9	1.22	0.51 – 2.92	0.92	0.37 – 2.29
132	Secondary Education Teachers	25	41	**1.88**	**1.13 – 3.11**	**1.76**	**1.05 – 2.94**	3	1.6	0.47 – 5.48	1.9	0.52 – 6.87
133	Teacher, Primary Teachers	36	28	0.86	0.52 – 1.43	0.79	0.47 – 1.32	1	0.43	0.06 – 3.11	0.44	0.06 – 3.56
139	Teachers n.e.c.	7	7	1.14	0.40 – 3.26	0.96	0.33 – 2.79	1	1.73	0.21 – 14.09	2.33	0.28 – 19.73
193	Social Workers	2	5	2.87	0.55 – 14.86	2.45	0.46 – 13.24	0		-		-
202	Memb Legislative Bodies, high civil servants	2	2	1.13	0.16 – 8.05	1.25	0.17 – 9.06	2	5.08	0.73 – 35.53	3.62	0.42 – 31.07
211	General Managers	12	12	1.15	0.51 – 2.57	1.16	0.51 – 2.65	3	2.05	0.58 – 7.32	2.82	0.68 – 11.69
212	Factory Managers	2	8	4.68	0.99 – 22.16	3.52	0.73 – 16.92	0		-		-
219	Managers n.e.c.	25	22	1.01	0.56 – 1.80	0.94	0.52 – 1.71	3	0.83	0.25 – 2.77	1.03	0.28 – 3.77
321	Secretaries, Typists, Stenographers	29	31	1.21	0.72 – 2.03	1.05	0.62 – 1.79	0		-		-
331	Bookkeepers, Cashiers	62	52	0.93	0.64 – 1.36	0.85	0.58 – 1.26	5	0.88	0.35 – 2.21	1.1	0.41 – 2.95
339	Financial Clerks	19	19	1.09	0.57 – 2.07	0.98	0.51 – 1.88	1	0.48	0.07 – 3.53	0.57	0.07 – 4.39
342	Computer Operators	2	7	3.94	0.82 – 19.09	3.27	0.66 – 16.25	0		-		-
370	Mail Distribution Clerks	28	16	0.64	0.34 – 1.19	0.63	0.33 – 1.18	2	0.58	0.14 – 2.39	0.6	0.14 – 2.63
391	Stockroom Attendants	19	16	0.95	0.49 – 1.86	0.84	0.42 – 1.66	4	1.69	0.57 – 5.02	1.95	0.62 – 6.14
393	Clerks	85	80	1.05	0.77 – 1.44	1.01	0.73 – 1.41	8	0.95	0.45 – 2.00	1.22	0.55 – 2.69
399	Clerks n.e.c.	3	6	2.34	0.58 – 9.41	2.44	0.59 – 10.09	0		-		-
410	Shop Keepers	64	41	0.71	0.47 – 1.06	0.70	0.46 – 1.05	6	0.69	0.27 – 1.73	0.66	0.25 – 1.76
421	Sales Managers	4	9	2.63	0.81 – 8.58	2.27	0.69 – 7.50	0		-		-
431	Sales Engineers	5	13	**3.01**	**1.07 – 8.49**	**3.02**	**1.05 – 8.66**	0		-		-
432	Traveling Salesmen	25	20	0.93	0.51 – 1.68	0.95	0.52 – 1.74	1	0.26	0.04 – 1.89	0.27	0.04 – 2.06
451	Sales Clerks	73	62	0.95	0.67 – 1.35	0.92	0.64 – 1.31	3	0.41	0.13 – 1.30	0.49	0.15 – 1.60
452	Newsvendors	27	21	0.89	0.50 – 1.58	0.87	0.48 – 1.57	2	0.56	0.14 – 2.31	0.48	0.11 – 2.06
500	Bar, Hotel Managers	21	14	0.77	0.39 – 1.53	0.83	0.41 – 1.68	3	0.73	0.21 – 2.55	0.65	0.18 – 2.43
510	Restaurant, Hotel Owners	22	12	0.62	0.31 – 1.27	0.66	0.32 – 1.35	2	0.92	0.22 – 3.90	1.12	0.26 – 4.92
531	Cooks	25	8	0.36	0.16 – 0.81	0.39	0.17 – 0.87	0		-		-
532	Waiters	27	26	1.08	0.63 – 1.87	1.03	0.59 – 1.80	4	1.37	0.47 – 3.98	1.07	0.33 – 3.46
540	Service Workers n.e.c.	56	41	0.79	0.52 – 1.21	0.82	0.53 – 1.26	1	0.31	0.04 – 2.16	0.31	0.04 – 2.22
551	Building Caretakers	23	10	0.49	0.23 – 1.03	0.46	0.22 – 0.99	1	0.38	0.05 – 2.71	0.28	0.04 – 2.16
552	Charworkers	55	56	1.13	0.76 – 1.66	1.17	0.79 – 1.74	2	0.61	0.15 – 2.52	0.73	0.17 – 3.10
560	Launderers	9	7	0.88	0.32 – 2.37	0.98	0.36 – 2.70	1	1.38	0.17 – 11.03	2.21	0.26 – 18.49
570	Hairdressers, barbers, etc.	17	8	0.52	0.23 – 1.22	0.52	0.22 – 1.23	0		-		-
580	Memb. Armed Forces n.e.c.	34	29	1.00	0.60 – 1.66	0.95	0.56 – 1.60	5	0.91	0.34 – 2.41	0.9	0.32 – 2.52
582	Policemen	28	18	0.72	0.40 – 1.32	0.66	0.36 – 1.22	2	0.45	0.11 – 1.83	0.34	0.07 – 1.56
589	Protective Service Workers n.e.c.	15	17	1.38	0.68 – 2.82	1.36	0.66 – 2.82	3	1.16	0.33 – 4.00	0.96	0.24 – 3.80
599	Other service workers	20	24	1.37	0.75 – 2.50	1.31	0.71 – 2.42	1	0.48	0.07 – 3.47	0.34	0.04 – 2.87
611	General Farmers	85	68	0.88	0.63 – 1.23	0.93	0.65 – 1.32	21	1.76	1.04 – 2.98	1.45	0.83 – 2.55
612	Specialized Farmers	36	48	1.54	0.99 – 2.39	**1.65**	**1.05 – 2.59**	7	1.06	0.46 – 2.42	0.94	0.39 – 2.25
621	General Farm Workers	262	218	0.93	0.76 – 1.14	1.04	0.83 – 1.31	37	0.94	0.63 – 1.43	0.69	0.44 – 1.09
622	Field Crop Workers	41	40	1.10	0.70 – 1.71	1.14	0.72 – 1.79	4	0.74	0.26 – 2.08	0.65	0.22 – 1.88
623	Palmwine Harvesters	27	27	1.14	0.67 – 1.97	1.16	0.67 – 2.01	6	1.35	0.55 – 3.35	1.45	0.57 – 3.68
624	Livestock Workers	42	32	0.84	0.52 – 1.35	0.89	0.54 – 1.44	14	**2.11**	**1.11 – 4.03**	1.58	0.79 – 3.16
625	Milkers	11	11	1.16	0.50 – 2.68	1.17	0.49 – 2.77	5	**3.64**	**1.22 – 10.83**	2.36	0.75 – 7.45
700	Foremen	27	26	1.11	0.64 – 1.91	1.07	0.61 – 1.89	2	0.47	0.11 – 1.94	0.69	0.16 – 2.95
711	Miners and Quarrymen	4	25	**7.34**	**2.54 – 21.20**	**7.96**	**2.72 – 23.23**	4	**4.98**	**1.23 – 20.18**	3.58	0.81 – 15.94
728	Galvinizers	4	8	2.32	0.69 – 7.73	2.51	0.74 – 8.49	2	4.08	0.74 – 22.47	5.42	0.92 – 31.95
729	Metal Processors n.e.c.	8	8	1.14	0.42 – 3.04	1.17	0.43 – 3.18	2	1.88	0.39 – 9.06	2.78	0.54 – 14.28
749	Chemical Workers	1	5	5.71	0.66 – 49.10	5.70	0.65 – 50.42	0		-		-
773	Butchers and Meat Preparers	17	4	0.27	0.09 – 0.81	0.26	0.09 – 0.78	4	1.74	0.58 – 5.28	1.86	0.54 – 6.34
774	Cannery Workers	12	15	1.40	0.65 – 3.02	1.61	0.74 – 3.53	2	1.96	0.43 – 8.93	2.28	0.47 – 11.18
776	Confectionery Makers	22	13	0.66	0.33 – 1.32	0.69	0.34 – 1.38	4	1.51	0.51 – 4.41	1.81	0.58 – 5.67
791	Tailors and Dressmakers	41	42	1.17	0.75 – 1.83	1.19	0.76 – 1.89	2	0.83	0.20 – 3.47	1.05	0.24 – 4.55
795	Sewing Machine Operators	24	22	1.06	0.59 – 1.93	1.06	0.58 – 1.94	1	0.69	0.09 – 4.99	0.5	0.06 – 4.14
796	Upholsterers	5	4	0.93	0.25 – 3.48	0.94	0.25 – 3.57	1	2.06	0.25 – 17.31	3.58	0.40 – 31.81
832	Tool and Die Makers	23	13	0.64	0.32 – 1.28	0.58	0.29 – 1.16	1	0.29	0.04 – 2.08	0.26	0.03 – 2.07
834	Machine Operators in Factory	59	50	0.96	0.65 – 1.42	0.94	0.63 – 1.39	5	0.72	0.29 – 1.81	0.75	0.29 – 1.96
839	Locksmiths	19	12	0.72	0.35 – 1.50	0.79	0.38 – 1.65	3	1.73	0.50 – 5.92	1.79	0.49 – 6.56
841	Machinists or Fitters	25	20	0.95	0.52 – 1.73	0.85	0.46 – 1.56	4	1.14	0.39 – 3.33	1.18	0.39 – 3.62
842	Instrument Makers	22	15	0.76	0.39 – 1.47	0.75	0.38 – 1.46	1	0.41	0.06 – 2.94	0.57	0.08 – 4.33
849	Machinery fitters, machine assemblers and precision instrument makers	45	40	1.01	0.65 – 1.56	0.98	0.63 – 1.52	7	1.42	0.63 – 3.23	2.08	0.88 – 4.92
855	Electricians	20	9	0.51	0.23 – 1.13	0.45	0.20 – 1.01	0		-		-
872	Welders	19	11	0.65	0.31 – 1.37	0.65	0.30 – 1.39	1	0.42	0.06 – 3.09	0.41	0.05 – 3.16
873	Sheet-Metal Workers	18	9	0.57	0.25 – 1.27	0.60	0.27 – 1.37	0		-		-
874	Structural Steel Workers	3	2	0.75	0.12 – 4.50	0.89	0.15 – 5.47	2	4.57	0.78 – 26.71	2.89	0.33 – 25.50
892	Potters	5	10	2.33	0.79 – 6.85	2.05	0.69 – 6.10	0		-		-
902	Tire Makers and Vulcanizers	7	3	0.49	0.13 – 1.90	0.60	0.15 – 2.35	2	2.78	0.58 – 13.38	3.91	0.73 – 20.98
910	Paper and paperboard product makers	8	13	1.87	0.77 – 4.55	1.72	0.69 – 4.25	3	2.34	0.61 – 8.95	2.47	0.59 – 10.41
922	Printing Pressmen	3	4	1.52	0.34 – 6.80	1.63	0.36 – 7.49	1	2.28	0.24 – 21.18	4.06	0.4 – 41.67
926	Bookbinders	3	7	2.59	0.67 – 10.06	2.53	0.64 – 10.08	0		-		-
931	Painters, Construction	16	18	1.31	0.66 – 2.58	1.23	0.61 – 2.46	3	2.06	0.58 – 7.23	1.59	0.41 – 6.23
942	Basketweavers	3	2	0.75	0.13 – 4.50	0.31	0.03 – 3.09	1	3.47	0.36 – 33.69	4.47	0.45 – 44.60
951	Masons	47	50	1.25	0.83 – 1.89	1.41	0.93 – 2.14	16	**2.41**	**1.33 – 4.36**	**2.55**	**1.36 – 4.78**
954	Carpenters	27	19	0.81	0.45 – 1.47	0.82	0.44 – 1.50	4	1.1	0.39 – 3.14	1.37	0.46 – 4.08
959	Construction Workers	16	10	0.70	0.32 – 1.56	0.76	0.34 – 1.7	7	**2.53**	**1.03 – 6.23**	**2.95**	**1.12 – 7.74**
969	Stationary engine and related equipment operators	6	3	0.59	0.15 – 2.37	0.73	0.18 – 2.97	4	**4.75**	**1.33 – 16.92**	**5.31**	**1.34 – 21.04**
971	Dockers and Freight Handlers	79	53	0.75	0.52 – 1.07	0.78	0.54 – 1.12	8	0.79	0.37 – 1.65	0.83	0.38 – 1.78
973	Crane and Hoist Operators	7	4	0.65	0.19 – 2.23	0.66	0.19 – 2.28	2	2.09	0.44 – 10.03	2.56	0.52 – 12.73
983	Railway Engine Drivers and Firemen	2	9	**5.08**	**1.09 – 23.65**	4.55	0.96 – 21.57	1	2.41	0.22 – 26.42	2.86	0.23 – 35.14
985	Drivers	64	53	0.95	0.65 – 1.39	0.95	0.65 – 1.40	8	0.85	0.40 – 1.79	0.79	0.37 – 1.73
999	Laborers n.e.c.	90	98	1.30	0.96 – 1.77	**1.39**	**1.01 – 1.89**	19	1.39	0.82 – 2.35	1.24	0.71 – 2.18

### All tumours (Table 3)

For the two histologic types taken jointly, the occupations that displayed an association with the disease were "Secondary education teachers" (OR 1.75, 95%CI 1.05–2.89, p-value = 0.03), "Miners and Quarrymen" (OR 7.04, 95%CI 2.44–20.31, p-value = 0.0003), "Masons" (OR 1.54, 95%CI 1.04–2.2, p-value = 0.032) and "Laborers" (OR 1.37, 95%CI 1.02–1.85, p-value = 0.04). Some occupations, such as "Cooks", "Building Caretakers", "Butchers and Meat Preparers", "Cabinetmakers", and "Electricians", registered a protective effect.

### Basal cell carcinomas (Table 4)

For BCC, excess risk was located in "Secondary education teachers" (OR 1.76, 95%CI 1.05–2.94, p-value = 0.03), "Sales Engineers" (OR 3.02; 95%CI 1.05–8.66, p-value = 0.04), "Specialized farmers" (OR 1.65, 95%CI 1.05–2.59, p-value = 0.03), "Miners and Quarrymen" (OR 7.96, 95%CI 2.72–23.23, p-value = 0.0002) and "Laborers" (OR 1.39, 95%CI 1.01–1.89, p-value = 0.04). "Railway Engine Drivers and Firemen" registered a significant increase in risk (OR 5.08, 95%CI 1.09–23.65), which subsequently lost significance on adjustment for sun and phenotype. The protective effect encompassed "Cooks", "Building Caretakers", and "Butchers and Meat Preparers".

### Squamous cell carcinomas (Table 4)

For SCC, the highest risk was detected in the occupations of "Construction worker" (OR 2.95, 95%CI 1.12–7.74, p-value = 0.03), "Stationary engine and related equipment operators" (OR 5.31, 95%CI 1.34–21.04, p-value = 0.02) and "Mason" (OR 2.55, 95%CI 1.36–4.78, p-value = 0.03, p-value = 0.004). In the case of "General farmers" (OR 1.76, 95%CI 1.04–2.98), "Livestock Workers" (OR 2.11, 95%CI 1.11–4.03), "Milkers" (OR 3.64, 95%CI 1.22–10.83) and "Miners and Quarrymen" (OR 4.98, 95%CI 1.23–20.18), there was an increase in risk on adjustment for age and sex, yet this rise in risk ceased to be significant when adjustment was subsequently made for solar exposure and phenotype. We detected no occupation with risk less than expected.

In the analysis of the three-digit occupations by center, the excess risk in miners was concentrated in Murcia, a province that accounted for 50% of all cases exposed, with an adjusted OR of 7.85 (95%CI, 1.57–39.26) among men. A possible high risk was also detected in Granada and Turin, but given the low frequency of this occupation, there was only one exposed control in each case. Analysis of miners by time of exposure yielded an OR of 3.62 (95%CI 1.01–13.16) for those who had worked as miners for less than 5 years, and 15.89 (95%CI 2.10–120.35) for those who had worked for 5 years or more (trend p-value 0.0008).

Dose-response analysis by time of exposure (no exposed, <5 years and 5 or more) for secondary education teachers, for masons and for laborers was statistically significant (trend p-value 0.01, 0.03 and 0.03 respectively).

In Besançon, the association was observed for secondary education teachers, with a risk of developing non-melanoma skin carcinoma of 3.23 (95%CI 1.02–10.23). Another center in which significant results were observed was Turin, with an OR -in this instance protective- of 0.10 (95%CI 0.01–0.76) for cooks.

## Discussion

This study analyzes the association between non-melanoma skin cancer and ISCO-coded occupations. Analysis of the major occupational groups showed that, in the context of basal cell carcinoma, professionals and technicians have an increased risk of developing this type of cancer. When all occupations and both histologic types were analyzed jointly, *miners and quarrymen*, *secondary education teachers *and *masons *registered excess risk. Separate analysis of the results by type showed a higher risk of basal cell carcinoma for *railway engine drivers and firemen*, *farmers *and *salesmen*, in addition to the above three occupations. The occupations that registered a higher risk of SCC (though not of BCC) were those involving *direct contact with livestock*, and the groups encompassing *other construction workers not elsewhere classified *(ISCO: 959) and *stationary engine and related equipment operators not elsewhere classified *(ISCO: 969).

This study include all incident cases registered in five of the participating centers that account for the 88% of the cases. This design prevent the existence bias based on occupational recruitment patterns. However, one possible source of bias could be the different population bases of the control sample; although a certain degree of distortion cannot be completely ruled out, consistency among centers was checked [8] and the country proved to be a stronger confounder than study design (hospital or population basis).

Multicenter studies such as this are an example of the indication of the use of mixed models. These models take the covariance structure or interdependence of data (characteristics not registered at each study center) into account, whereas fixed effects models assume that all observations are independent. The ensuing estimates and standard errors may possibly be more conservative, but the inferences that can be drawn from the results are wider.

A major problem of this type of exploratory study is that a large number of studied associations could produce some spurious significant results, the so-called mass-significance phenomenon. In order to deal with the problem of multiple comparisons, p-values are provided in results section. The number of statistically significant associations found exceed very little the results expected by chance, but we consider that these results in addition to the dose-response effect with exposure time for some of the occupations, could stimulate the research about the influence of occupational exposures on this tumours.

One of the aims of this analysis was to assess the risk due to exposures other than solar radiation, yet adjustment for solar radiation and phenotype did not substantially modify the effect estimates (Tables 3 and 4). Some of the associations detected were in outdoor occupations (construction workers or farmers); exposure to sun is inherent in such occupations and may thus account for the fact that adjustment has scant influence on the result. However, in the case of other occupations for which an effect was detected, such as mining, possible explanations must be sought elsewhere.

Relatively few studies have addressed occupation and exposures other than solar radiation, in the case of these tumours. In NMSC, the role of exposure to various chemical substances has been reported. Elevated risks of squamous cell carcinoma have been detected among subjects exposed to pesticides and by-products of petroleum, lubricants and other substances. In the case of basal cell carcinoma, higher risks have also been documented in subjects exposed to fiberglass dust and dry-cleaning agents [15], though stress has nonetheless been laid on the greater importance of exposure to arsenic versus other chemical substances in the etiology of these tumours [28].

It has also been reported that 2% of such tumours could be associated with exposure to radon in the UK. [29]. The results of our study show a strong association between the occupation of miner and both histologic types of NMSC, with the strength of association for BCC being double that for SCC. The explanation for this result might partly lie in the above-mentioned exposure to radon in the case of BCC [30]; and possibly lie in exposure to arsenic in the case of SCC [13,28]. However, a rise in risk of precancerous skin lesions has been reported among workers in open-work lignite mines, a finding that could be attributable to the long-term increase in the risk of skin cancer [31]. The OR estimations shown wide confidence intervals, reflecting some data instability and we can not discard the effect of uncontrolled confounders.

Although this type of cancer has not been shown to be more frequent in specific social groups [32], the association between NMSC and ionizing radiations has indeed been described on a number of occasions [33-36] and is reputedly greater with BCC than with SCC [29,34]. Occupational exposure to UV radiation among outdoor workers has a direct relationship with the appearance of these types of tumours [37-40]. In our study, farmers/animal husbandry workers were observed to register an increased risk of developing both BCC and SCC, despite our efforts to adjust for exposure to solar radiation. It is well known, however, that farmers suffer from multiple exposures [41], ranging from pesticides to hazardous air pollutants (HAPs), due to their use of different types of machinery and plants. The raised risk of basal cell carcinoma among railway engine drivers and firemen has been reported in other studies [19]. Though somewhat rare, it is acknowledged that occupational skin cancer can appear in the case of scars formed as a consequence of industrial burns [21].

The results show that the possible confounding effect generated by such solar exposure is very small, since OR magnitudes varied very little after this variable was adjusted for. Phenotype likewise failed to modify risk levels, with adjustment for it leading to no important variations vis-à-vis the crude effect. Moreover, we do not know the magnitude of the residual confounding effect of solar exposure. However, our questionnaire, for skin characteristics measurements and reported sun exposure history, received a validation study and there was a good reproducibility [42].

Among workers in direct contact with livestock, risk is apparently higher for SCC. Although there is a slight possibility of false diagnoses of SCC in the case of viral warts, such a problem would seem unlikely, in view of the fact that the cases were reviewed by a panel of pathologists who verified the diagnoses. These results evinced a high degree of concordance (99.5%), with a Kappa index (KI) of 0.85 (95%CI 0.77–0.94) in the assessment of the malignancy of lesions. Concordance in the differentiation of major morphologic groups, BCC and SCC was also high (KI = 0.85; 95%CI 0.82–0.89) [24]. There is limited evidence in humans for the carcinogenicity of HPV genus-beta types in skin (squamous-cell carcinoma). In the rare case of patients with epidermodysplasia verruciformis, there is compelling evidence for the carcinogenicity of HPV genus-beta types 5 and 8 in skin (squamous-cell carcinoma)[43].

## Conclusion

This study shows the association between non-melanoma skin cancer and certain occupations. For NMSC as a whole (both *histologic types*), miners and quarrymen, secondary education teachers, and masons register excess risk, regardless of exposure to solar radiation and phenotype. BCC proves more frequent among railway engine drivers and firemen, farmers and salesmen, in addition to the above-mentioned 3 occupations. The occupations that register a higher risk of SCC (though not of BCC) are those involving direct contact with livestock, other construction workers not elsewhere classified and stationary engine and related equipment operators not elsewhere classified. Exposure to HAPs, arsenic, ionizing radiations and burns might well explain a good part of the associations observed in this study. The Helios Project affords an excellent opportunity for further in-depth study based on matrices of occupational exposure.

## Competing interests

The author(s) declare that they have no competing interests.

## Authors' contributions

BS and GLA performed the statistical analysis and wrote the first draft of the manuscript to which all authors subsequently contributed. RZ, SR, CM, CN, MJT, HSG, JW, LG, SS were responsible for the development of intellectual content and the multicenter study design and conducting. All authors made contribution to statistical analyses and interpretation of results, and revised the manuscript for important intellectual content. All authors read and approved the final manuscript.

## Pre-publication history

The pre-publication history for this paper can be accessed here:


